# Differential timing and latitudinal variation in sex ratio of Aquatic Warblers during the autumn migration

**DOI:** 10.1007/s00114-017-1525-x

**Published:** 2017-11-14

**Authors:** Katarzyna Wojczulanis-Jakubas, Małgorzata E. Chrostek, Frédéric Jiguet, Carlos Zumalacárregui Martínez, David Miguélez, Júlio M. Neto

**Affiliations:** 1 Department of Vertebrate Ecology and Zoology, University of Gdańsk, ul. Wita Stwosza 59, 80-308 Gdańsk, Poland; 2 Centre d’Ecologie et des Sciences de la Conservation, UMR 7204 MNHN-CNRS-UPMC-Sorbonne Universités, CP 135, 55 Rue Buffon, 75005 Paris, France; 3 Fundación Global Nature, Corro Postigo, 1, 34337 Palencia, Fuentes de Nava Spain; 4 Iberian Ringing Group (GIA-León), C/Daoiz y Velarde, 49 bajo, 24006 León, Spain; 5 Departament of Biodiversity and Environmental Management, University of León, Campus de Vegazana s/n, 24071 León, Spain; 6 CIBIO/UP - Centro de Investigação em Biodiversidade e Recursos Genéticos, Universidade do Porto, Campus Agrário de Vairão, Rua Padre Armando Quintas, 4485-661 Vairão, Portugal; 7 Molecular Ecology & Evolution Lab, MEMEG, Department of Biology, University of Lund, Ecology Building, Sölvegatan 37, SE-223 62 Lund, Sweden

**Keywords:** *Acrocephalus paludicola*, Migration strategy, Sex proportion

## Abstract

Differential migration has been extensively reported in spring, but less so in autumn, particularly in relation to sex in monomorphic bird species. Here, we analysed the autumn passage of a monomorphic, globally threatened passerine, the Aquatic Warbler *Acrocephalus paludicola* throughout Western Europe, with regard to age and sex. We showed that, overall, adults migrated earlier than first-year birds, and males migrated earlier than females during the autumn migration. This may be caused by an overall social dominance of adults over immatures, and differentiated migration strategy of males and females. In addition, we found male-skewed sex proportions, with a tendency to an equalised ratio in more southern stopover sites. This may indicate a male bias in the global population or different migration strategies of the sexes. Differential migration may cause the age and sex classes to be exposed differently to various threats affecting demographic structure of the species.

**Electronic supplementary material:**

The online version of this article (10.1007/s00114-017-1525-x) contains supplementary material, which is available to authorized users.

Differential migration occurs when distinguishable classes of individuals, usually age and sex categories, differ with respect to timing, route or destination of the migration (Newton 2011). It has been demonstrated in many species from various phylogenetic and ecological groups that adults and first-year birds tend to migrate at different times. Differential sex-related avian migration has been extensively studied in spring but little attention has been paid to autumn migration (Mills 2005; Jakubas and Wojczulanis-Jakubas 2010). Differential migration in autumn may be attributed to ecological, physiological, social factors and/or the differential distribution of sex classes throughout the migration routes and wintering range (Newton 2011).

Here, we study the autumn migration of a small monomorphic passerine, the Aquatic Warbler *Acrocephalus paludicola*, with regard to age and sex. This is a globally threatened species of sedge meadows in continental Europe that global population declined by 90% during the last century (Birdlife International 2015). A basic knowledge about important events such as the migration period is essential for efficient conservation management of the global population. Northern populations of the Aquatic Warbler migrate to sub-Saharan Africa (Flade et al. 2011) through Western Europe, using stopover sites in marshes mostly in France, Spain and Portugal (Julliard et al. 2006; Miguélez et al. 2009; Neto et al. 2010). Only one study has examined the relation between sex, age and timing of autumn migration (Wojczulanis-Jakubas et al. 2013). Albeit being limited to a single study year and single study site, that study found earlier migration of adults males compared to adult females and first-years birds of both sexes. A male-biased sex ratio was also found in that study. To explore whether these results apply to the species on a larger spatial and temporal scale, we analyse here the pattern of autumn migration regarding age and sex at different stopovers along the Western European migration route.

## Methods

### Bird capture

**Fig. 1:**
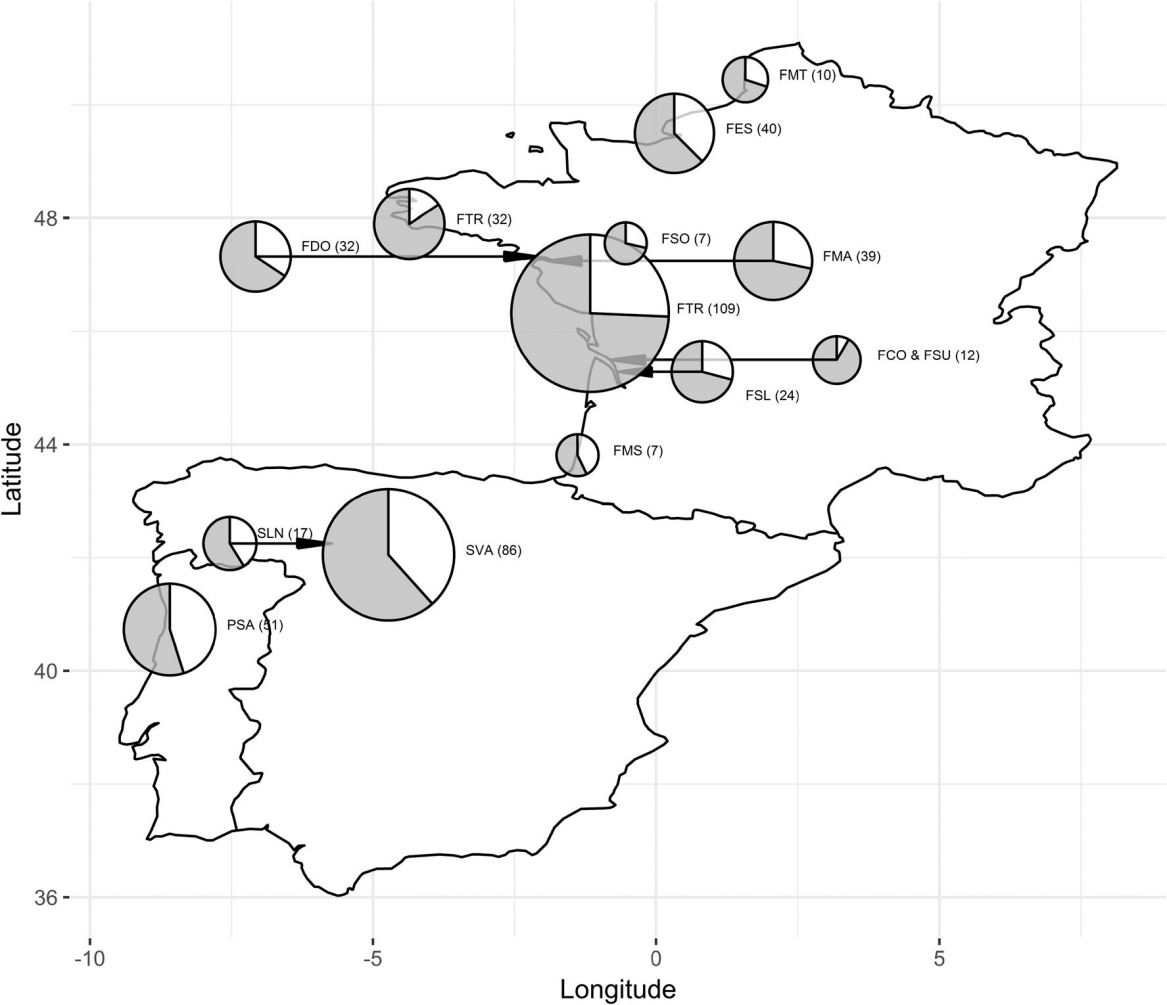
Proportions of captured males (grey) and females (white) of the Aquatic Warbler (*Acrocephalus paludicola*; both age combined) over the stopover sites, with pie centres located at the stopovers. For clarity of the picture, five pies were moved slightly eastward or westward and connected with the right coordinates with the arrows. The pie size denotes relative sample size. The stopover name code (Table 1) and the number of birds captured (in brackets) set at the right-site of corresponding pie

We captured the birds along a wide latitudinal gradient, from 50.44^o^ N to 40.43^o^ S, at 14 stopover sites (Fig. 1, Table 1). We used similar capturing protocol across all study sites (Table 1; slight differences in the protocol between the sites are unlikely to have an impact on the number and dynamics of the captures). We captured the birds using mist-nets placed in and along reedbeds. At each site, we opened the mist-nets ca 30 min before local sunrise and kept them operating for 5–6 h. We used diurnal playback (of the Aquatic Warbler’s song) to attract the birds to the mist-netting area across all the sites. This was because Aquatic Warblers are difficult to capture if not stimulated by playback (Julliard et al. 2006) and playback was used in the study of Wojczulanis-Jakubas et al. (2013), which results we aimed to verify. Although playback is known to bias age and/or sex ratio (e.g. Lecoq and Catry 2003), the effect is not consistent across species, including *Acrocephalus* genus (e.g. Wojczulanis-Jakubas et al. 2015). Our data indicate that song playback does not affect the proportions of the age and sex categories of the Aquatic Warbler. The only playback effect we found is that it tends to attract leaner birds, which are presumably recent arrivals looking for suitable foraging areas to replenish their energy stores (Supplementary materials – Effect of playback: Fig. S1 and Fig. S2, Table S1). Importantly, playback was used consistently at all study sites. In total, at each site we operated for at least 25 days of the migration period, capturing on average 33 birds per site (range 4–109; Table 1).

**Table 1:** Capturing characteristic in the 14 stopover sites across the Western Europe. The asterisks indicate sites pooled due to spatial proximity

Country	Site	Site code	Lat	Long	Capturing schedule	*N* days of capturing	Onset of capturing (Julian day)	End of capturing (Julian day)	Adult females	Adult males	First-year females	First-year males	Total *n* birds
France	Merlimont	FMT	50.446	1.574	Interrupted	32	208	245	0	0	3	7	10
	EstuaireSeine	FES	49.498	0.321	Daily	39	207	245	1	6	14	19	40
	Trunvel	FTR	47.896	−4.36	Interrupted	71	196	273	1	2	4	25	32
	Soulaire	FSO	47.554	−0.541	Daily	37	209	245	0	0	2	5	7
	Donges	FDO	47.321	−2.075	Daily	52	200	251	4	8	7	13	32
	Massereau	FMA	47.244	−1.933	Daily	32	213	244	2	4	9	24	39
	Triaize	FTR	46.32	−1.167	Daily	39	212	254	8	29	20	52	109
	Saint Seurind Uzet*	FSU	45.499	−0.834	Interrupted	26	218	264	0	0	1	3	4
	Conchemarche*	FCO	45.485	−0.785	Interrupted	50	198	258	0	3	0	5	8
	Braud SaintLouis	FSL	45.285	−0.686	Interrupted	25	217	250	1	2	6	15	24
	Messanges	FMS	43.81	−1.39	Interrupted	30	228	273	0	0	3	4	7
Spain	La Nava (2 seasons)	SLN	42.25	4.73	Interrupted	49–50	212–214	258–262	12	22	21	31	86
	Valcavado	SVA	42.05	5.73	Interrupted	31	214	245	5	8	2	2	17
Portugal	Salreu (5 seasons)	PSA	40.73	−8.594	Interrupted	24–33	213–216	259–273	10	15	13	13	51

*Sites pooled due to spatial proximity (57 km apart) and the low sample size for each

We ringed all captured individuals and aged them based on plumage. To sex the birds, we used molecular techniques and/or discriminant functions proposed by Jakubas et al. 2014 (see details in Supplementary Materials). For purpose of the molecular sexing, we collected 3–6 body feathers (samples from France), a single tail feather (Spain) or a drop of blood (Portugal). To sex the birds with the discriminant function, we measured wing length using a ruler with 1 mm accuracy. We captured 471 birds of which 95% individuals were molecularly sexed, 5% were sexed using the discriminant functions (all from Spain), and only five birds were not sexed due to missing DNA sample and measurements (those were excluded from analyses).

### Data analysis

Since no significant inter-annual variation in Julian days of passage was observed for the sites where the fieldwork was carried out for more than one season (Portugal, seasons 2009–2013: LM, *F*_4,49_ = 0.92, *P* = 0.46; Spain, seasons 2005–2007: lm, *F*_1,84_ = 0.08, *P* = 0.78; Table 1), we pooled data of all seasons for a given site. We also pooled data from two close neighbouring stopover sites in France (separated by only 57 km) that had relatively small sample size (Table 1).

To find the best model to examine the phenology of autumn migration in regard to age and sex across the latitudinal gradient, we generated a set of models with combination of age, sex, latitude (continuous predictor) and interactions and evaluated them using *dredge* function in MuMIn package (Bartoń 2016). Of that we chose the model with the lowest value of Akaike criteria and the highest weight (Table S2), and that included all predictors and only single, age and sex interaction (24% of the variance in the day of passage explained, *F*_4,461_ = 38.67, *P* < 0.001). We boxcox-transformed Julian day before the analysis.

We examined the variation in sex proportions in each age category separately using binomial test. We also analysed probability of males capturing using logistic regression (*logit* function). Again, to find the best model, we generated a set of models with combination of the age, latitude and their interactions and chose the one with the lowest value of Aikake criteria (Table S3). The final model included only the latitude.

Since we sexed some birds (all from Spain) using discriminant function and that could potentially affect the results, we performed all analyses on two data sets, with and without the individuals sexed based on morphology. We obtained qualitatively similar results for the full and restricted data set, therefore we concluded that the effect of the sexing method on the findings is negligible and proceeded with full data set. We performed all analyses in R environment (version 3.3.3, R Core Team 2017).

## Results

Day of passage differed significantly between age categories (*lm*, *F*_1,461_ = 64.44, *P* < 0.001), with adults preceding the first-year birds by 6.7 days (*SE* = 1.6; *t*_461_ = −4.45, *P* < 0.001). The day of passage also differed between the sexes (*lm*, *F*_*1*,461_ = 12.55, *P* < 0.001), with males preceding the females by 3.8 days (*SE* = 1.6; *t*_461_ = 2.54, *P* = 0.01). Finally, the day of passage changed with latitude (*lm*, *F*_*1*,461_ = 75.44, *P* < 0.001), with significant shift of the passage by 1.2 day (*SE* = 0.2) per one degree of latitude (*t*_461_ = 8.66, *P* < 0.001). We did not reveal significant difference in the timing of passage for particular age and sex categories (insignificant interaction of age and sex; *lm*, *F*_*1*,461_ = 2.26, *P* < 0.001).

Overall, the proportion of the sexes was male biased, both in adults (0.69, 95% CI 0.61–0.77; *P* < 0.001), and first-year birds (0.67, 95% CI 0.62–0.73, *P* < 0.001). However, we found a tendency to more equal proportions in southern stopover sites (Fig. 1). This tendency was apparent while modelling the probability of males capturing, when we found that the odds of males capturing slightly but significantly decreased with the latitude (by 1.1 per one degree of latitude, *SE* = 0.04; glm, *Z* = 2.40, *df* = 464, *P* = 0.01).

## Discussion

Adults passing earlier than first-year birds during the autumn migration have been reported in numerous species (reviewed in Newton 2011), including the Aquatic Warbler (e.g. Miguélez et al. 2009; Neto et al. 2010). Such a pattern seems to be typical for the long-distant migrants that undergo the moulting on the wintering ground (Newton 2011), as the Aquatic Warbler is. The most commonly evoked explanation of the observed pattern is that adults, being more experienced and socially dominant over the first-year birds may orientate better and may also better optimise the time of migration, and so migrate faster (Newton 2011).

The protandrous pattern of autumn migration seems to be uncommon in passerines (Mills 2005; Newton 2011), and difficult to explain. Since autumn migration starts after the breeding is completed (Newton 2011), and in the Aquatic Warbler that differs for males and females (only females look after the young in the Aquatic Warbler, Cramp 1998), one could expect adult males departing earlier than adult females and first-year birds of both sexes, as found in a single site and season (Wojczulanis-Jakubas et al. 2013). Here, however, examining the issue over a large geographical scale, we found that the differential migration of the two sexes was independent of age (insignificant interaction of sex and age). One possible explanation of the protandrous pattern observed in the present study could be that males, might be socially dominant over females (Newton 2011), and as such could efficiently defend the best feeding patches and fuel up more rapidly than females. As such, the males might migrate faster and arrive earlier to the stopover sites than females.

We found that the overall number of captured males exceeded the number of females. We did not find evidence that usage of playback could affect the proportions of the age and sex categories in the Aquatic Warbler thus, the finding reflects real differences in sex ratios between the sites/latitudes. Various reasons may account for the pattern observed (e.g. biased overall sex ratio in the population, alternative migration route more frequently used by females). Importantly, the finding suggests that the sexes can be exposed differently to various threats, what in turn may affect demographic structure of the species.

## Electronic supplementary material

ESM 1 (DOCX 85 kb)

## References

[CR1] Bartoń K (2016) Multi-Model Inference. R package version 1.15.6. https://CRAN.R-project.org/package=MuMIn

[CR2] Birdlife International (2015). European red list of birds.

[CR3] Cramp S (1998) The complete birds of the Western Palearctic on CDROM. Oxford University Press, Software©Optimedia

[CR4] Flade M, Diop I, Haase M, Le Névé A, Oppel S, Tegetmeyer C, Vogel A, Salewski V (2011). Distibution, ecology and threat status of the Aquatic Warblers (*Acrocephalus paludicola*) wintering in west Africa. J Ornithol.

[CR5] Jakubas D, Wojczulanis-Jakubas K (2010). Sex- and age-related differences in the timing and body condition of migrating Reed Warblers *Acrocephalus scirpaceus* and Sedge Warblers *Acrocephalus schoenobaenus*. Naturwissenschaften.

[CR6] Jakubas D, Wojczulanis-Jakubas K, Foucher J, Dziarska-Pałac J, Dugue H (2014). Age and sex differences in fuel load and biometrics of aquatic warblers *Acrocephalus paludicola* at an autumn stop-over site in the Loire estuary (NW France). Ardeola.

[CR7] Julliard R, Bargain B, Dubos A, Jiguet F (2006). Identifying autumn migration routes for the globally threatened Aquatic Warbler *Acrocephalus paludicola*. Ibis.

[CR8] Lecoq M, Catry P (2003). Diurnal tape-luring of wintering chiffchaffs results in samples with biased sex ratios. J Field Ornithol.

[CR9] Miguélez D, Zumalacárregui C, Fuertes B, Astiárraga H, González-Jáñez R, Roa I, de la Calzada F (2009). Habitat, phenology and biometrics of the aquatic warbler *Acrocephalus paludicola* during autumn migration through a riverine wetland in Iberia. Ring Migr.

[CR10] Mills AM (2005). Protogyny in autumn migration: do male birds “play chicken”?. Auk.

[CR11] Neto JM, Encarnação V, Fearon P (2010). Distribution, phenology and conditions of aquatic warblers *Acrocephalus paludicola* migrating through Portugal. Ardeola.

[CR12] Newton I (2011). Migration within the annual cycle: species, sex and age differences. J Ornithol.

[CR13] R Core Team (2017) R: a language and environment for statistical computing. R https://www.r-project.or

[CR14] Wojczulanis-Jakubas K, Jakubas D, Foucher J, Dziarska-Pałac J, Dugué H (2013). Differential autumn migration of the aquatic warbler *Acrocephalus paludicola*. Naturwissenschaften.

[CR15] Wojczulanis-Jakubas K, Wietrzykowski J, Jakubas D (2015). Response of Reed Warbler and Sedge Warbler to acoustic playback in relation to age, sex, and body condition. J Ornithol.

