# Supplementary material for: Differential timing and latitudinal variation in sex ratio of Aquatic Warblers during the autumn migration
Source: Naturwissenschaften. 2017 Nov 14;104(11):101. doi: 10.1007/s00114-017-1525-x (PMC5686252; doi:10.1007/s00114-017-1525-x)
Supplement: Supplementary file 1 — (DOCX 85 kb) [file 114_2017_1525_MOESM1_ESM.docx]

**Supplementary materials:** *Differential timing and latitudinal variation in sex ratio of Aquatic Warblers during the autumn migration.*

**Effect of playback**

The effect of playback usage on the capturing rates in the Aquatic Warbler *Acrocephalus paludicola* was tested during autumn migration in Portugal (single ringing site; seasons between 1977 and 2013; two groups of treatment: with and without stimuli) and France [33 ringing sites; season 2013; two groups of treatment: with strong (playback at all mist nets) and weak acoustic stimuli (playback at few mist nets)].

An equal proportions of the two age groups were found in both playback-treatment groups in Portugal (X^2^ = 0.03, n = 122, P = 0.85, Fig S1a). Also, no differences in the proportion of adults and first-year birds were found in France; in both playback-treatment groups more first-year birds than adults were captured (X^2^ = 0.01, n = 356, P = 0.91, Fig S1b).

**
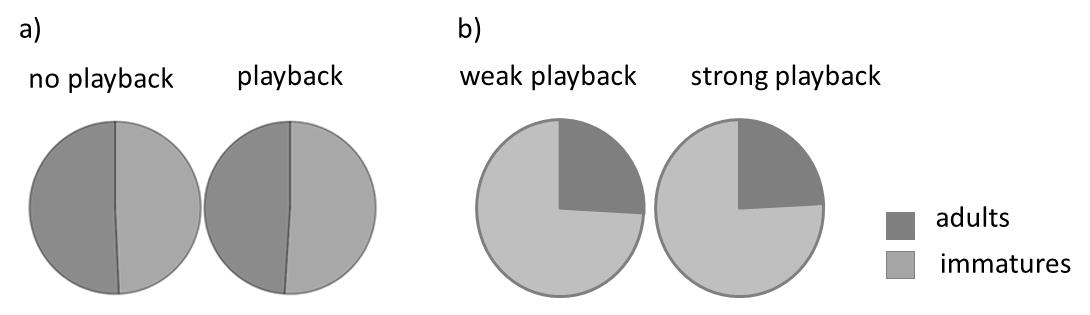
**

**Figure S1**. Effect of song playback on the age of Aquatic Warblers trapped during the fieldwork a) in Portugal, and b) in France.

Male-biased sex ratio was observed in the two playback-treatment groups in France, both in adults (X^2^ = 0.18, n = 87, P = 0.67; Fig S2a) and first-year birds (X^2^ = 0.02, n = 269, P = 0.89; Fig S2b).


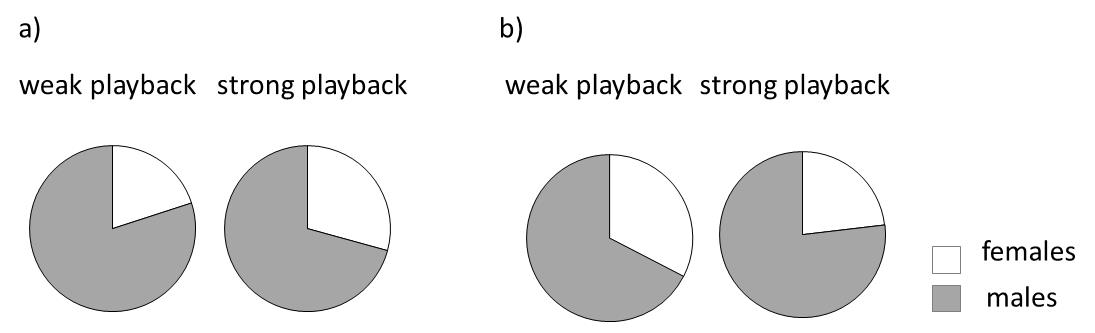


**Figure S2**. Effect of strong (applied at all mist nest) and weak playback (applied at few mist nets) on the sex in a) adult and b) first-year Aquatic Warblers trapped in France.

The only significant effect of playback was found when examining the birds body mass in Portugal. It was found that leaner birds tended to be attracted to the mist-nets stronger when the playback was applied (Table S1).

**Table S1**. Parameter estimates, t-tests and effect sizes of a general linear model (R^2^ = 0.19; n = 105) determining the effect of playback (with = 1; without = 0) on the body mass of Aquatic Warblers captured in Portugal between 1977 and 2013; controlled for the effects of day of capture (1 = 1^st^ August), age (first-year birds; adults) and wing length.

| Parameter | B | Std. Error | t | Sig. | 95% Conf Interval | | Partial Eta Squared |
| --- | --- | --- | --- | --- | --- | --- | --- |
|  |  |  |  |  | Lower Bound | Upper Bound |  |
| Intercept | 1.778 | 6.682 | 0.266 | 0.791 | -11.478 | 15.035 | 0.001 |
| Wing | 0.135 | 0.105 | 1.279 | 0.204 | -0.074 | 0.344 | 0.016 |
| Day | 0.031 | 0.013 | 2.494 | **0.014** | 0.006 | 0.056 | 0.059 |
| [Age= first-year birds] | 0.277 | 0.349 | 0.793 | 0.430 | -0.415 | 0.968 | 0.006 |
| [Age=adults] | 0 |  |  |  |  |  |  |
| [Playback=0] | 0.867 | 0.296 | 2.925 | **0.004** | 0.279 | 1.455 | 0.079 |
| [Playback=1] | 0 |  |  |  |  |  |  |

**Model choice**

**Table S2**. Ranking of models (ordered by value of AICc ) in analysis of Julian day of passage.

Global model formula (LM, with Gaussian error distribution): Julian day of passage (boxcox-transfomed) ~ Age * Sex * Latitude

| Intercept | Age | Latitude | Sex | Age * Latitude | Age * Sex | Latitude * Sex | Age * Latitude * Sex | df | AICc | delta | weight |
| --- | --- | --- | --- | --- | --- | --- | --- | --- | --- | --- | --- |
| 1.02E-02 | + | 2.04E-04 | + |  | + |  |  | 6 | -11240.7 | 0.00 | 0.236 |
| 1.04E-02 | + | 2.05E-04 | + |  |  |  |  | 5 | -11240.4 | 0.23 | 0.210 |
| 1.15E-02 | + | 1.75E-04 | + | + | + |  |  | 7 | -11239.3 | 1.38 | 0.119 |
| 1.10E-02 | + | 1.86E-04 | + |  | + | + |  | 7 | -11239.0 | 1.70 | 0.101 |
| 1.14E-02 | + | 1.83E-04 | + | + |  |  |  | 6 | -11238.8 | 1.90 | 0.091 |
| 1.07E-02 | + | 1.99E-04 | + |  |  | + |  | 6 | -11238.4 | 2.24 | 0.077 |
| 1.25E-02 | + | 1.51E-04 | + | + | + | + |  | 8 | -11237.7 | 2.97 | 0.053 |
| 1.04E-02 | + | 2.11E-04 |  |  |  |  |  | 4 | -11237.0 | 3.65 | 0.038 |
| 1.17E-02 | + | 1.75E-04 | + | + |  | + |  | 7 | -11236.8 | 3.90 | 0.034 |
| 1.07E-02 | + | 1.92E-04 | + | + | + | + | + | 9 | -11236.2 | 4.50 | 0.025 |
| 1.13E-02 | + | 1.91E-04 |  | + |  |  |  | 5 | -11235.3 | 5.35 | 0.016 |
| 1.91E-02 | + |  | + |  | + |  |  | 5 | -11172.5 | 68.19 | 0.000 |
| 1.94E-02 | + |  | + |  |  |  |  | 4 | -11172.2 | 68.49 | 0.000 |
| 1.97E-02 | + |  |  |  |  |  |  | 3 | -11164.3 | 76.37 | 0.000 |
| 1.21E-02 |  | 1.45E-04 | + |  |  |  |  | 4 | -11152.0 | 88.64 | 0.000 |
| 1.21E-02 |  | 1.44E-04 | + |  |  | + |  | 5 | -11150.0 | 90.68 | 0.000 |
| 1.20E-02 |  | 1.52E-04 |  |  |  |  |  | 3 | -11147.6 | 93.09 | 0.000 |
| 1.86E-02 |  |  | + |  |  |  |  | 3 | -11121.6 | 119.11 | 0.000 |
| 1.89E-02 |  |  |  |  |  |  |  | 2 | -11114.0 | 126.68 | 0.000 |

**Table S3**. Ranking of models (ordered by value of AICc ) in analysis of probability of males capturing.

Global model formula (GLM, with binomial family and logit function): Sex ~ Age * Latitude.

| Intercept | Age | Latitude | Age * Latitude | df | AICc | delta | weight |
| --- | --- | --- | --- | --- | --- | --- | --- |
| -30.530 |  | 0.08431 |  | 2 | 582.3 | 0.00 | 0.489 |
| -33.110 | + | 0.09349 |  | 3 | 583.3 | 1.01 | 0.295 |
| -42.250 | + | 0.11430 | + | 4 | 585.2 | 2.91 | 0.114 |
| 0.7550 |  |  |  | 1 | 586.1 | 3.80 | 0.073 |
| 0.8109 | + |  |  | 2 | 587.9 | 5.68 | 0.029 |

**Methods - sexing Aquatic Warblers**

We stored the samples until molecular analyses in ambient temperature in individual plastic or paper bags (feathers) or in 70% ethanol (blood samples). We extracted DNA from feathers using a kit for forensic samples, Sherlock AX (A&A Biotechnology, Gdynia, Poland), obtaining on average 8.72 mg/μl of DNA with purity estimated on average on 1.44 (A260/280). A standard salt extraction method was used to extract DNA from the blood samples, which were then diluted to 5ng/μl. We performed molecular sexing on feather-based DNA using a standard PCR-based method, with a ready-to-use PCR-mix with *Taq* polymerase (REDTaq® ReadyMix™ PCR Reaction Mix, Sigma Aldrich, USA; samples from France) or Multiplex PCR Kit (Quiagen, Germany; samples from Spain). Initially, we used P2/P8 primers, targeting introns on CHD-1 gene (Griffiths et al. 1998), following protocols used to sex the Aquatic Warbler by Wojczulanis-Jakubas et al. (2013). However, the efficiency of amplification with P2/P8 was low (31%; samples from France), and so we processed the rest of the samples with F2/R1 pair of primers following Bantock et al. (2008) protocol, resulting in c.99% efficiency for samples from France and 79% efficiency for samples from Spain. These primers target introns in ATP5A1 – alpha subunit of mitochondrial ATP synthase gene that is a highly conservative unit of the genome (Bantock et al. 2008). The sex difference in the introns length in PCR products analysed with both pair of primers was clearly visible when separated on agarose gel (3.5%) stained with Midori Green Advanced DNA (Nippon Genetics, Japan) and viewed in UV-light. For blood-based DNA, we performed PCR with the Z002 primer pair (Dawson 2007) following the procedures described by Neto et al. (2011). All blood-based samples were sexed successfully.

For birds which were not feather or blood sampled, or for which the material collected was insufficient to successfully amplify the target DNA region, we used a discriminant function to determine the sex (Jakubas et al. 2014). We applied separate functions for adults (D_adults_ = wing length × 0.757 – bill depth × 5.009 – 31.889), and first-year birds (D_first-year birds_ = wing length × 0.824 – 52.473). The function has 87% and 75% efficiency in correct sexing of adults and first-year aquatic warblers, respectively (Jakubas et al. 2014). Since we did not measure bill depth, one of the two measurements in the function for adults, we used the average value for adults migrating through France (3.02 mm) from Jakubas et al. (2014).

**Literature cited:**

Bantock TM, Prys-Jones RP, Lee PLM (2008) New and improved molecular sexing methods for museum bird specimens. Mol Ecol Res 8:519–528

Dawson DA (2007) Genomic analysis of passerine birds using con- served microsatellite loci. Ph.D. thesis. University of Sheffield

Griffiths R, Double MC, Orr K, Dawson RJG (1998) A DNA test to sex most birds. Mol Ecol 7:1071–1075

Jakubas D, Wojczulanis-Jakubas K, Foucher J, Dziarska-Pałac J, Dugue H (2014) Age and sex differences in fuel load and biometrics of aquatic warblers *Acrocephalus paludicola* at an autumn stop-over site in the Loire estuary (NW France). Ardeola 61:15–30

Neto JM, Hansson B, Hasselquist D (2011) Sex allocation in Savi’s Warblers *Locustella luscinioides*: multiple factors affect seasonal trends in brood sex ratios. Behav Ecol Sociobiol 65:297–304

Wojczulanis-Jakubas K, Jakubas D, Foucher J, Dziarska-Pałac J, Dugué H (2013) Differential autumn migration of the aquatic warbler *Acrocephalus paludicola*. Naturwissenschaften 100:1095–1098
